# Role of CCT4/ErbB signaling in nephroblastoma: Implications for a biomarker of Wilms tumor

**DOI:** 10.1097/MD.0000000000033219

**Published:** 2023-04-14

**Authors:** Haoyuan Wang, Lei Zhang, Bin Liu, Jianzhi Su, Xiaochen Ni

**Affiliations:** a Department of Urology Surgery, The Fourth Hospital of Hebei Medical University, Shijiazhuang, Hebei, PR China; b Department of Urology Surgery, Fuxing Hospital Affiliated to Capital Medical University, Xicheng District, Beijing, PR China.

**Keywords:** bioinformatics, biomarker, CCT4, WGCNA, Wilms tumor

## Abstract

Wilms tumor is a common abdominal malignant tumor in children. However, the molecular mechanism of Wilms tumor is unclear. GSE66405 and GSE197047 were obtained from the Gene Expression Omnibus database. To identify differentially expressed genes (DEGs) in Wilms tumor, the R package “limma” was used. Weighted gene co-expression network analysis was performed to identify the significant module. The list of DEGs was input into the Search Tool for the Retrieval of Interacting Genes database to construct a protein-protein interaction network for predicting core genes. Gene Ontology analysis and the Kyoto Encyclopedia of Genes and Genomes analysis are computational methods for assessing gene function and biological pathways. The genome was analyzed by Gene Ontology and Kyoto Encyclopedia of Genes and Genomes and developed by gene set enrichment analysis. Comparative Toxicogenomics Database analysis was performed to find the diseases most related to the core genes. TargetScan was used to screen for miRNAs that regulate hub genes. A total of 925 DEGs were identified. The differently expressed genes were mainly enriched in the metabolic pathway, AMPK signaling pathway, ErbB signaling pathway, mRNA detection pathway, and folded protein binding. A total of 16 core genes (HNRNPK, PABPC1, HNRNPD, NCL, YBX1, EIF4G1, KHDRBS1, HNRNPAB, HSPA4, EEF2, HSP90AA1, EEF1A1, A TP5A1, SDHA, CCT4, CCT5) were obtained. chaperonin containing TCP-1 subunit 4 (CCT4) was downregulated in tumor tissue samples, which may have reverse regulatory significance for Wilms tumor. CCT4, HSP90AA1, NCL, PABPC1, and YBX1 were found to be associated with kidney disease, acute kidney injury, edema, tumor metastasis, transitional cell carcinoma, necrosis, and inflammation. The research found that the related miRNA of the CCT4 gene was hsamiR-7-5p. CCT4 might play an essential role in the occurrence and development of Wilms tumor, and they may participate in the occurrence and development of Wilms tumor through the ERBB signal pathway. CCT4 may be a promising biomarker of Wilms tumor.

## 1. Introduction

Wilms tumor is a common abdominal malignant tumor in children, accounting for 90% of renal tumors in children aged 0 to 14 years.^[1]^ Clinically, it is also called renal embryoma, which originates in the kidney, with abdominal mass as the first symptom, and the incidence is increasing year by year.^[2,3]^ The combination of surgery, radiotherapy, and chemotherapy has dramatically progressed in treating Wilms tumor. The 5-year overall survival rate is 90%, but there are still patients with poor prognosis and recurrence.^[4,5]^ However, for a small number of children with nephroblastoma, the disease continues to progress after treatment, and some of them even have a recurrence, and the prognosis is not good.^[6,7]^ Wilms tumor develops from renal cells without mature differentiation and has a certain tendency of familial occurrence. However, the exact etiology is still unclear. Therefore, it is imperative to study the molecular mechanism of nephroblastoma.

With the development of second-generation sequencing, methods based on bioinformatics emerged.^[8,9]^ Bioinformatics is a new interdisciplinary subject in biological science, and its research focus is mainly reflected in genomics and proteomics. It uses modern information technology to simulate and predict protein structure and drug design based on the analysis of many data, such as DNA sequencing and functional genome. Weighted gene co-expression network analysis (WGCNA) is a method to calculate the tumor purity and core module of tumor tissue. Gene expression profile^[10,11]^ calculates the core molecular target of tumor tissue.

Chaperonin containing TCP-1 (CCT) complex is a molecular chaperone protein that exists widely in the cytoplasm. About 10% of the cytoplasmic proteins, including actin and tubulin, are folded by CCT.^[12]^ Actin and tubulin are involved in the occurrence and development of tumors, such as cell division, migration, and invasion. As a subunit of the CCT complex, CCT subunit 4 (CT4) also plays a role in different tumors. For example, CCT4 gene amplification was detected in clinical lung cancer cases, low expression of CCT4 was associated with decreased survival, suggesting a poor prognosis, and changes in the expression of CCT4 in cancer cells could significantly affect cell migration.^[13]^ There was differential expression of CCT4 at the mRNA level in cancer tissues. In tumors, CCT can regulate tumor progression by folding a variety of tumor suppressor genes and other cytokines, such as VHL, P53, ErbB, CDC20, and STAT3.^[14–17]^ As a subunit of the CCT complex, CCT4 regulates these genes. Although CCT4 is abnormally expressed in various tumors and regulates multiple cancer-related genes, its expression, and role in nephroblastoma are still unclear.

Therefore, this paper intends to use bioinformatics technology to mine the core genes between nephroblastoma and normal tissue and carry out enrichment analysis and pathway analysis. Typical data sets were used to verify the significant role of CCT4 in nephroblastoma. And it was verified by a primary cell experiment. It is expected to provide a new direction for the molecular mechanism of Wilms tumor.

## 2. Methods

### 2.1. Wilms tumor data set

In this study, the tumor dataset GSE66405 and GSE197047 configuration files were generated and downloaded from GPL17077 and GPL24676 Gene Expression Omnibus database (http://www.ncbi.nlm.nih.gov/geo/). Gene Expression Omnibus is a public functional genomics data repository that accepts array-based and sequence-based data and provide tools to help users query and download experimental and curated gene expression profiles. GSE66405 included 28 Wilms and 4 standard tissue samples, and GSE197047 included 8 Wilms and 8 standard tissue samples to identify differentially expressed genes (DEGs) in Wilms.

### 2.2. Batch removal treatment

We first used the R software package (Ross Ihaka and Robert Gentleman, University of Auckland, New Zealand) to merge and de-batch multiple data sets to merge GSE66405 and GSE197047. For the conformation of multiple data sets, we first used the R software package in Silico Merging [DOD:10.1186/1471-2105-13-335] to merge data sets and get a merge matrix. Further, we used the remove Batch Effect function of the R software package limma (version 3.42.2) to remove the batch effect. Finally, after the batch effect removal, we obtained the matrix and applied it to the subsequent analysis.

### 2.3. Screening of DEGs

The R package “limma” was used for probe summary and background correction of the merge matrix for GSE66405 and GSE197047. The Benjamini–Hochberg method was used to adjust the original *P* value. The false discovery rate (FDR) was used to calculate fold change. The cutoff standard for DEG is FDR < 0.05. And make the volcano map, and take intersection DEGs with the Venn diagram.

### 2.4. WGCNA

WGCNA aims to find co-expressed gene modules and explore the association between gene networks, interest phenotypes, and the network’s core genes. First, we calculated each gene’s median absolute deviation using the post-batch removal confluence matrix of GSE66405 and GSE197047. The first 50% of median absolute deviation minimal genes were excluded, and the outlier genes and samples were removed using the suitable Samples Genes method of the R software package WGCNA. Further, WGCNA was used to construct the scale-fresco-expression network. To be specific, first, for all pairs, gene performs the Pearson correlation matrix and average chain method, then, using the power function A_mn = | C_mn | ^ beta structure weighted adjacency matrix (C_mn = Gene_m and Pearson correlation between Gene_n; A_mn = adjacency between Genem and Genen). *β* is a soft threshold parameter, which can emphasize the strong correlation between genes and weaken the influence of weak correlation and negative correlation. After selecting a power of 10, the adjacency is converted into a topological overlap matrix (TOM), which can measure the network connectivity of a gene, defined as the sum of its adjacency with all other genes, used for the network gene ratio, and the corresponding dissimilarity (1-TOM) is calculated. To classify genes with similar expression profiles into gene modules, average linkage hierarchical clustering was performed according to the TOM-based anisotropy measure, and the minimum size of the gene tree graph (genome) was 30. Set the sensitivity to 3. To further analyze the modules, we calculated the heterogeneity of the characteristic genes of the modules, selected a cut line for the module tree, and incorporated some modules. It is worth noting that gray modules are considered collections of genes that cannot be assigned to any module.

### 2.5. Construction and analysis of protein-protein interaction (PPI) network

The Search Tool for the Retrieval of Interacting Genes (STRING) is a database of known and predicted PPIs. STRING database (https://cn.string-db.org/cgi/input.pl) is designed to collect, store, and integrate all publicly available sources of information on PPIs and to supplement these sources with computational predictions. In this study, the list of differential genes was input into the STRING database to build a PPI network for predicting core genes (confidence > 0.4). Cytoscape software (U.S. National Institute of General Medical Sciences) can provide biologists with biological network analysis and 2-dimensional (2D) visualization. In this study, Cytoscape software was used to visualize and predict the core genes of the PPI network formed by a STRING database. First, the PPI network was imported into Cytoscape software, and the modules with the best correlation were found through MCODE. In addition, 2 algorithms (MCC and MNC) were used to calculate the 10 genes with the best correlation respectively and take intersection. After visualization, the list of core genes was derived.

### 2.6. Functional enrichment analysis

Gene Ontology (GO) analysis and Kyoto Encyclopedia of Genes and Genomes (KEGG) analysis are computational methods for assessing gene function and biological pathways. This study will help determine the difference between gene list input KEGG rest (https://www.kegg.jp/kegg/rest/keggapi.html) to obtain the latest KEGG pathway gene annotation. With this as the background, the genes were mapped to the background set, and the enrichment analysis was performed using the R software package cluster Profiler (version 3.14.3) to obtain the enrichment results of the gene set. The GO annotation of genes in the R software package org.Hs.e.g..db (version 3.1.0) was also used as the background to map the genes into the background set. The minimum gene set was 5, and the complete gene set was 5000. A *P* value of <0.05 and an FDR of <0.25 were considered statistically significant measures.

In addition, the Metascape database provides a comprehensive resource for annotating and analyzing gene lists with visual export. We use the Metascape database (http://metascape.org/gp/index.html) in the line of the above differences in gene enrichment of function analysis and export list.

### 2.7. Gene set enrichment analysis (GSEA) analysis

GSEA is a computational method used to determine whether an a priori-defined set of genes is statistically significant with significant, consistent differences between 2 biological states. For GSEA (DOI: 10.1073/ pnas. 0506580102, http://software.broadinstitute.org/gsea/index.jsp) web station received GSEA software (version3.0), divided the sample into 2 groups based on Wilms tumor and normal tissue, and derived from Molecular Signatures the Database (DOI: 10.1093/bioinformatics/btr260, http://www.gseamsigdb.org/gsea/downloads.jsp) to download C2. Cp. Kegg. V7.4. Symbols. To review the related pathways and molecular mechanisms of grouping based on gene expression and phenotype, Gene Matrix Transposed collections set the minimum gene sets 5, the biggest gene sets for 5000, 1000 heavy samplings, *P* value < 0.05, and FDR < 0.25 were considered statistically significant. The whole genome was analyzed by GO and KEGG and developed by GSEA.

### 2.8. Gene expression heat map

We used an R-packet heatmap to make a heatmap of the expression levels of core genes found by the 2 algorithms in the PPI network in GSE66405 and GSE197047 to visualize the expression differences of core genes between Wilms tumor and standard tissue samples.

### 2.9. Comparative Toxicogenomics Database (CTD) analysis

CTD is a robust publicly available database that provides manually curated information on chemo-gene/protein interactions, chemo-disease, and gene-disease relationships. CTD integration of many chemicals, genetic and functional data, and the interactions between the phenotype and disease-related environmental exposure factors for disease and drug potential mechanism research provides excellent convenience. We input the core genes into the CTD website to find the diseases most related to the core genes and use Excel (Microsoft, Redmond, WA) to draw the radar map of the expression difference of each gene.

### 2.10. miRNA

TargetScan was used to predict miRNA binding sites. TargetScan (www.targetscan.org) is an online database for the predictive analysis of miRNA and target genes. Our study used TargetScan to screen for miRNAs that regulate central DEG.

## 3. Results

### 3.1. Differential gene analysis

**Figure 1.**
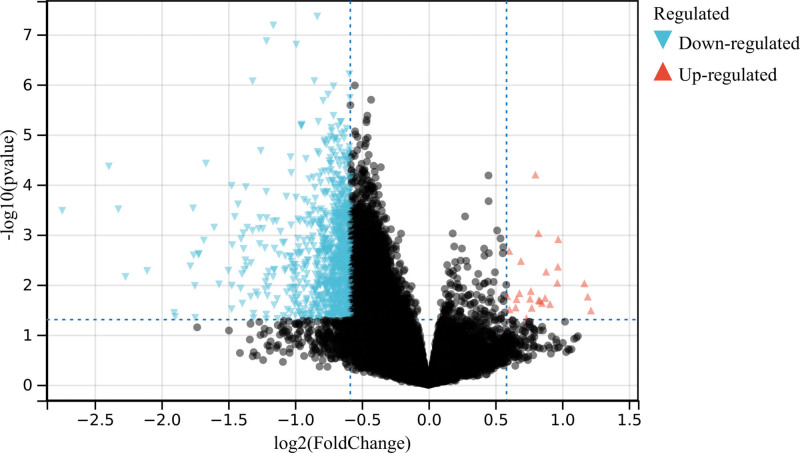
A total of 925 DEGs were identified according to the batch removal merge matrix of GSE66405 and GSE197047. DEGs = differentially expressed genes.

In this study, 925 DEGs were identified according to the batch removal merge matrix of GSE66405 and GSE197047 (Fig. 1).

### 3.2. Functional enrichment analysis

#### 3.2.1. Functional enrichment analysis of DEGs.

**Figure 2.**
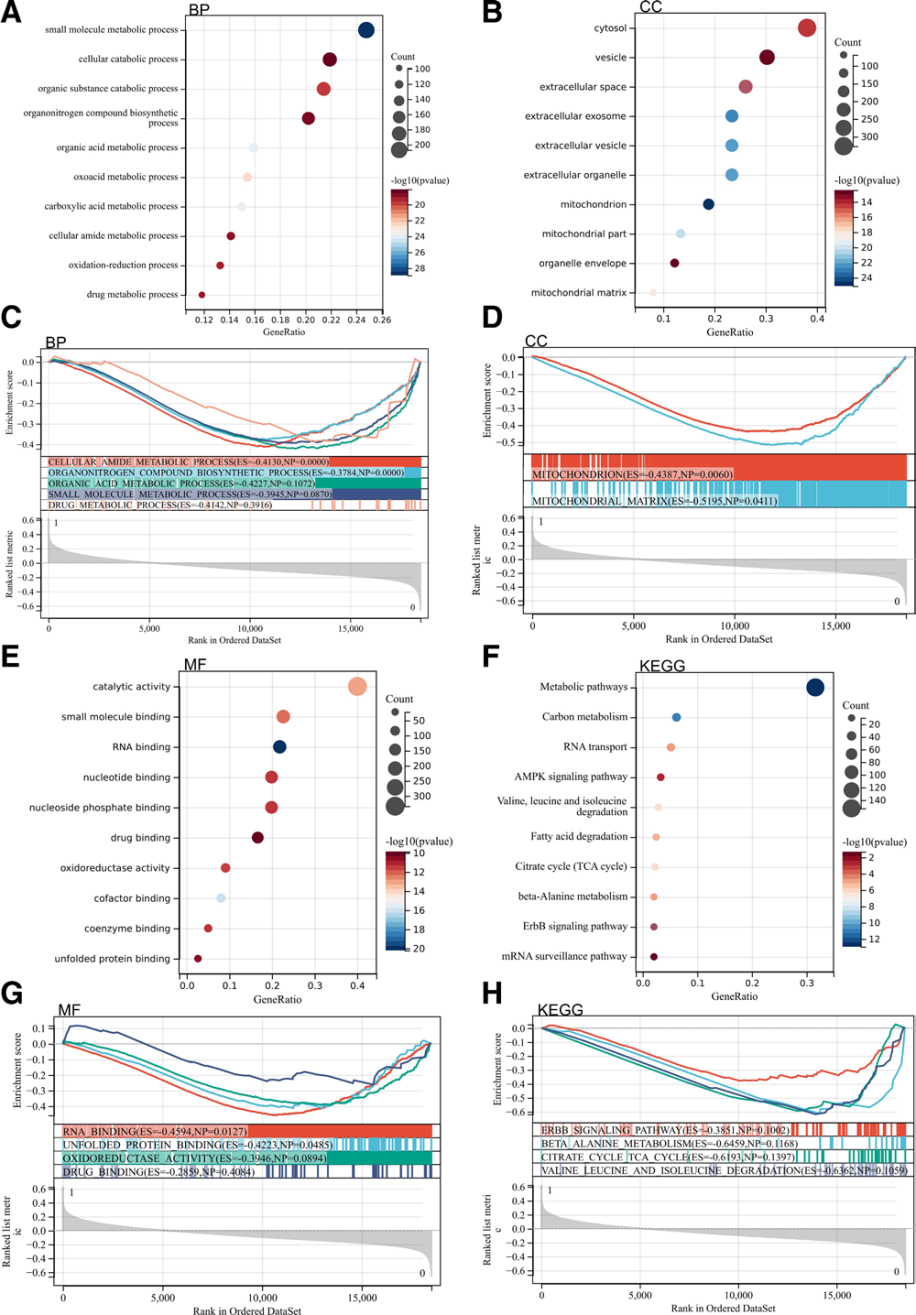
Functional enrichment analysis for the differently expressed genes. And they were mainly enriched in the metabolic pathway, AMPK signaling pathway, ErbB signaling pathway, mRNA detection pathway, and folded protein binding.

Then we performed GO and KEGG analysis on these DEGs. According to GO analysis, they were mainly enriched in the metabolic pathway, AMPK signaling pathway, ErbB signaling pathway, mRNA detection pathway, and folded protein binding (Fig. 2A, C, E, and G).

### 3.3. GSEA analysis

In addition, GSEA enrichment analysis was conducted on the whole genome to find possible enrichment items in non-DEGs. The results are shown in the figure. The enrichment items were similar to the GO and KEGG enrichment items of DEGs, mainly enriched in the folding protein binding and ErbB signaling pathway (Fig. 2B, D, F, and H).

### 3.4. Metascape enrichment analysis

**Figure 3.**
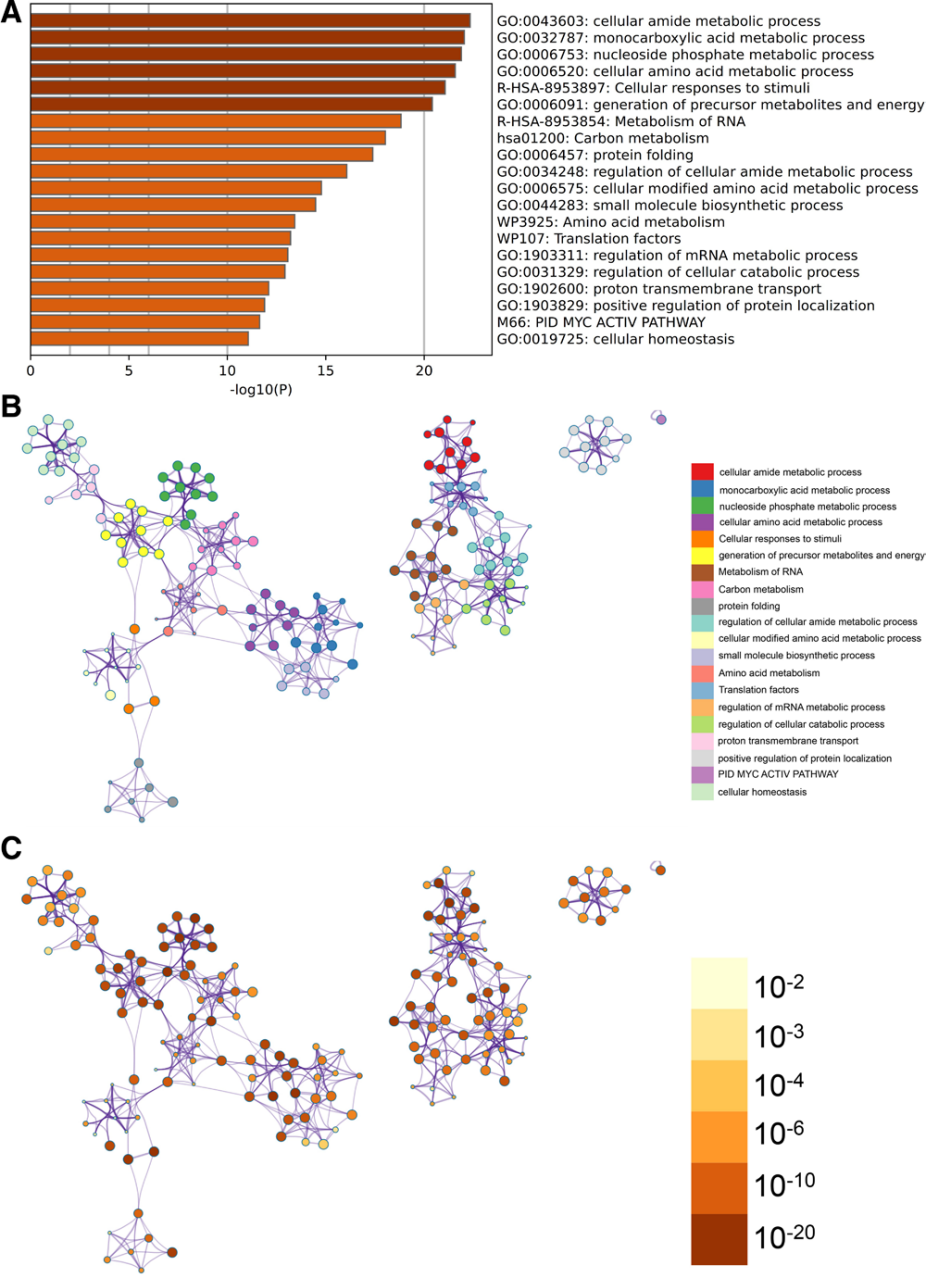
Metascape enrichment analysis.

In the enrichment project of metascape, GO has a positive regulation of protein localization, protein folding (Fig. 3A), and enrichment networks with enrichment term coloring and *P* value coloring (Fig. 3B and C).

### 3.5. WGCNA analysis

**Figure 4.**
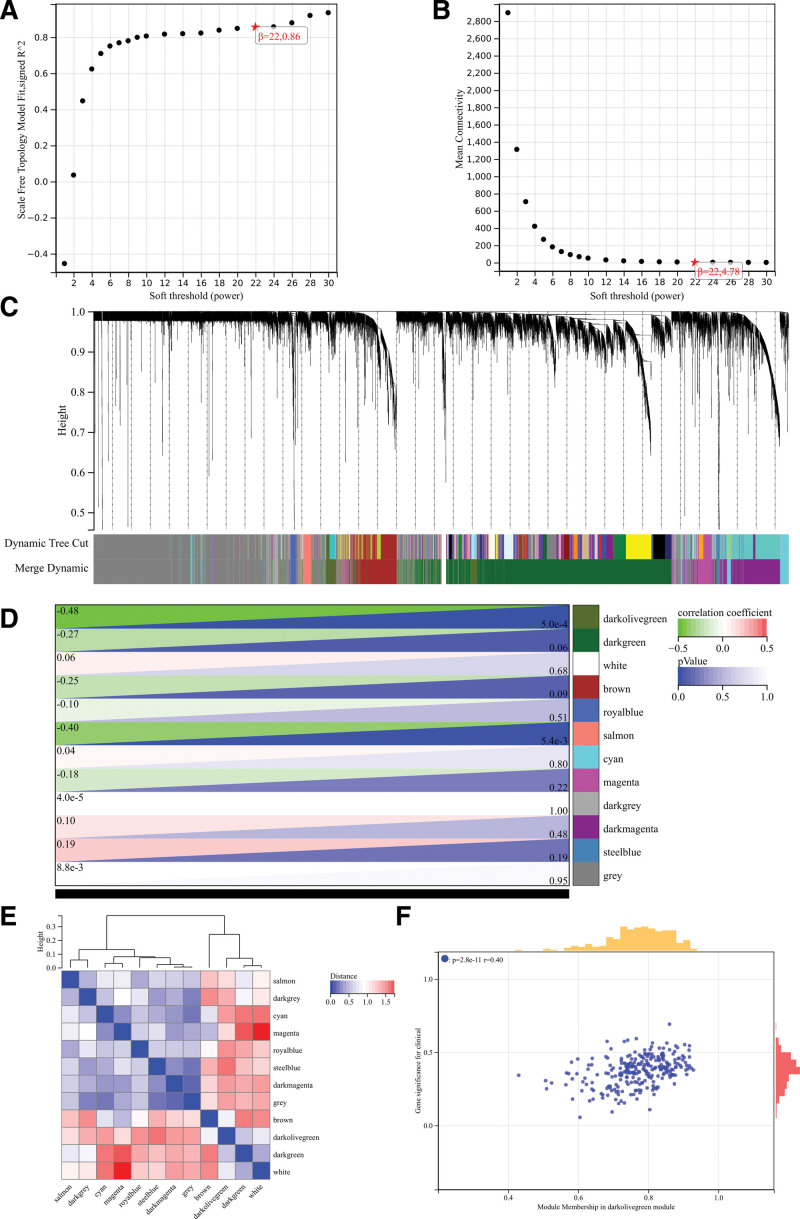
WGCNA analysis. (A and B) Soft threshold power in the WGCNA analysis. (C) Hierarchical cluster trees of all genes. (D) Correlation heat map between modules and phenotypes. (E) The interactions between these modules. (F) Correlation scatters map between GS and MM of related hub genes. GS = gene significance, MM = module membership, WGCNA = weighted gene co-expression network analysis.

Soft threshold power selection is an essential step in WGCNA analysis. Perform network topology analysis to determine the soft threshold power. The soft threshold power in the WGCNA analysis was set to 9, the lowest power for the scale-free topological fitting index of 0.9 (Fig. 4A and B). Hierarchical cluster trees of all genes were constructed, and necessary modules were generated (Fig. 4C). The correlation heat map between modules and phenotypes was presented (Fig. 4D). The interactions between these modules are then analyzed (Fig. 4E). And the correlation scatters map between gene significance and module membership of related hub genes was generated (Fig. 4F).

### 3.6. Construction and analysis of PPI network

**Figure 5.**
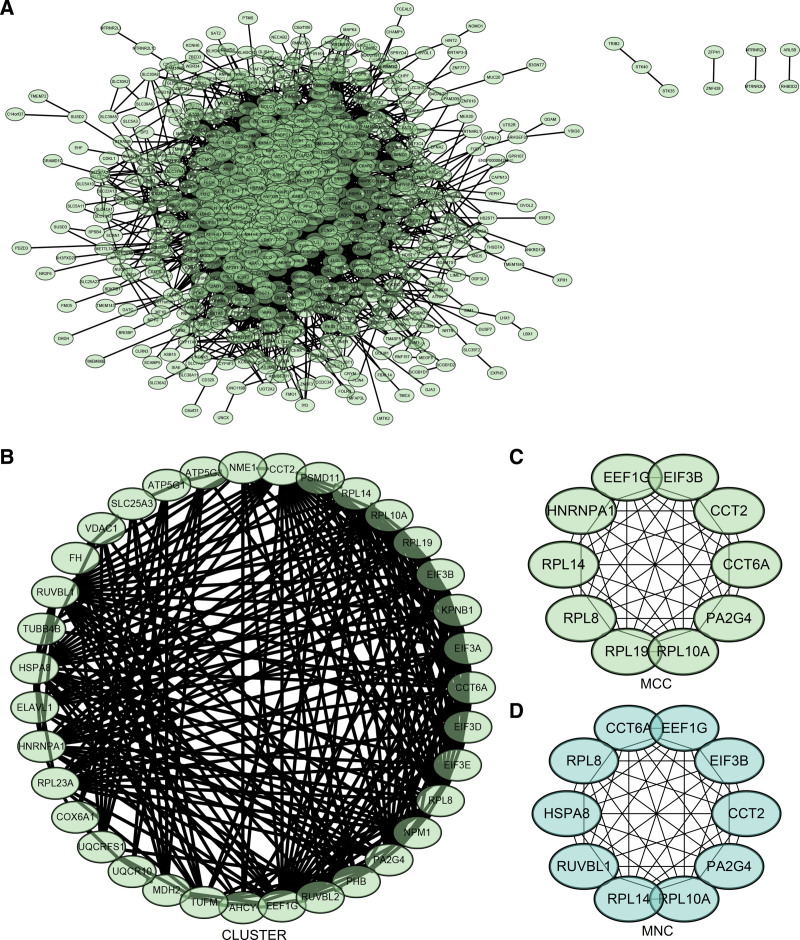
Construction and analysis of protein-protein interaction (PPI) network. (A) The STRING online database constructed the PPI network of DEGs. (B) Hub genes. (C and D) Two different algorithms were used to identify the central genes. DEGs = differentially expressed genes, STRING = Search Tool for the Retrieval of Interacting Genes.

The PPI network of DEGs was constructed by the STRING online database and analyzed by Cytoscape software (Fig. 5A). The core gene cluster (Fig. 5B) was obtained, and 2 different algorithms were used to identify the central genes (Fig. 5C and D). Sixteen core genes (HNRNPK, PABPC1, HNRNPD, NCL, YBX1, EIF4G1, KHDRBS1, HNRNPAB, HSPA4, EEF2, HSP90AA1, EEF1A1, A TP5A1, SDHA, CCT4, CCT5) were obtained.

### 3.7. Gene expression heat map

**Figure 6.**
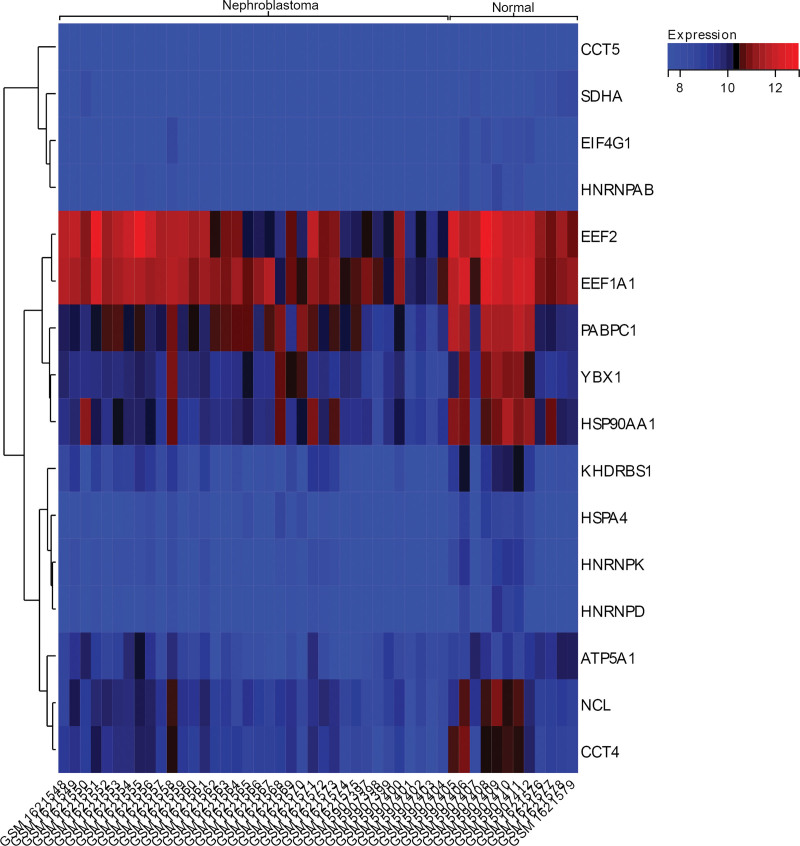
The heat map of the difference in expression of core genes between Wilms tumor and standard tissue samples.

We visualized the heat map of the difference in expression of core genes between Wilms tumor and standard tissue samples (Fig. 6). We found that core genes (CCT4, HSP90AA1, NCL, PABPC1, YBX1) were lowly expressed in tumor tissue samples and highly expressed in standard tissue samples, which may have reverse regulatory significance for Wilms tumor.

### 3.8. CTD analysis

**Figure 7.**
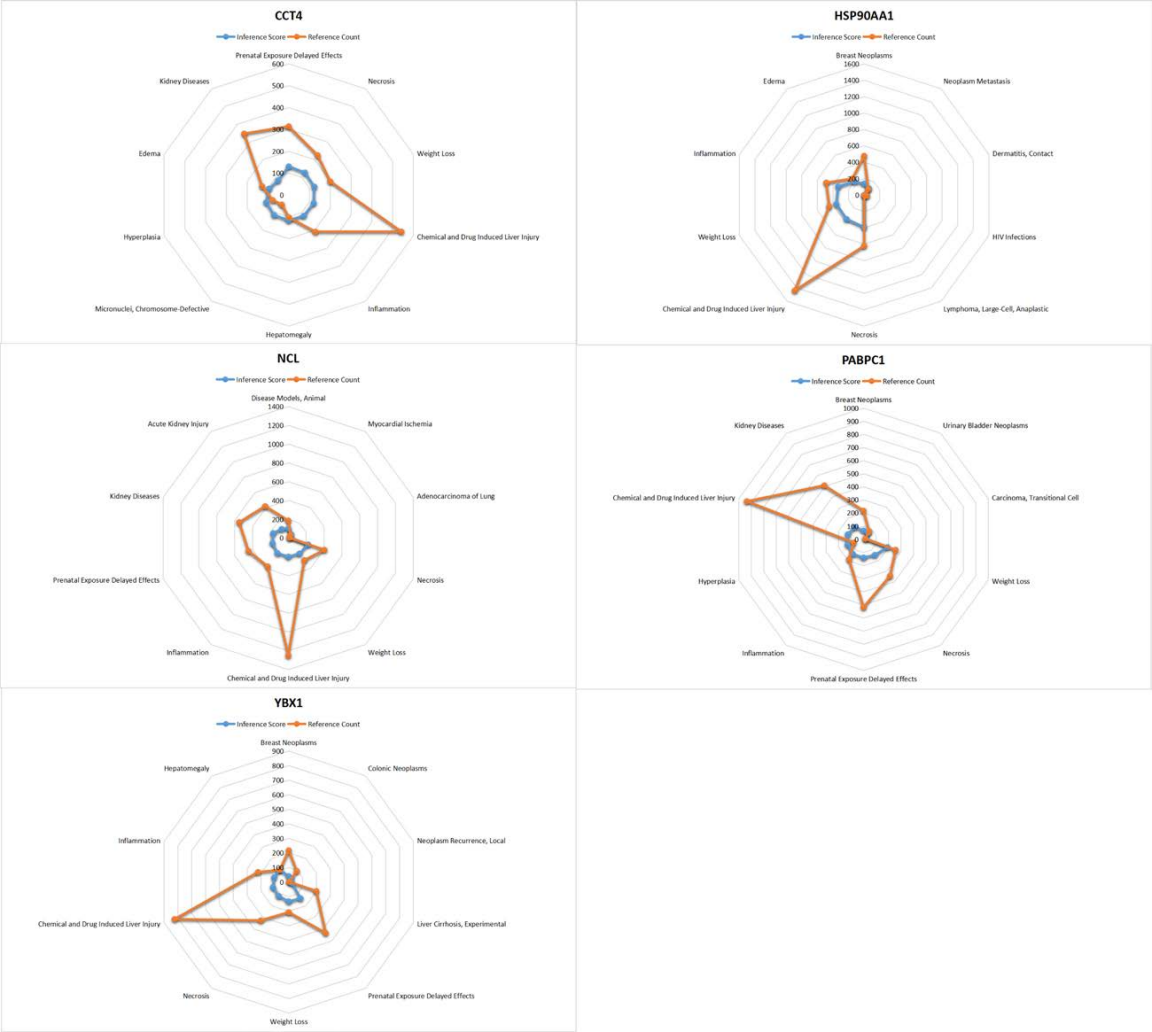
Four genes (CCT4, HSP90AA1, NCL, PABPC1, YBX1) were associated with kidney disease, acute kidney injury, edema, tumor metastasis, transitional cell carcinoma, necrosis, and inflammation.

In this study, we entered the hub gene list into the CTD website to search for diseases related to the core genes, improving the understanding of the gene and disease association. Five genes (CCT4, HSP90AA1, NCL, PABPC1, YBX1) were found to be associated with kidney disease, acute kidney injury, edema, tumor metastasis, transitional cell carcinoma, necrosis, and inflammation (Fig. 7).

### 3.9. Prediction and functional annotation of miRNA associated with hub gene

In this study, the hub gene list was input into TargetScan to search for related miRNA and improve the understanding of gene expression regulation (Table 1). We found that the related miRNA of the CCT4 gene was hsamiR-7-5p; the related miRNA of the HSP90AA1 gene was hsa-miR-9-5p; the related miRNA of the NCL gene was hsa-miR-194-5p; the related miRNA of PABPC1 gene were hsa-miR-129-5p and hsa-miR-1295p. The related miRNA of the YBX1 gene is hsa-miR-137.

**Table 1:** A summary of miRNAs that regulate hub genes.

	Gene	miRNA	
1	CCT4	hsa-miR-7-5p	
2	HSP90AA1	hsa-miR-9-5p	
3	NCL	hsa-miR-194-5p	
4	PABPC1	hsa-miR-129-5p	hsa-miR-129-5p
5	YBX1	hsa-miR-137	

miRNA = microRNA.

## 4. Discussion

Wilms tumor is the most common embryonic malignant tumor in children. Tumorigenesis is a multi-step and complex process related to the uncontrolled growth of tumor cells. Upregulation of oncogene expression and downregulation of tumor suppressor gene expression during the growth of a tumor can result in malignant development. The study of targeted drugs needs to deeply explore the molecular mechanism of Wilms tumor. The main result of this study is that the expression of CCT4 is low in nephroblastoma. When CCT4 is activated, the ERBB signal pathway is inhibited, the apoptosis is enhanced, the expression of MMP3/9 is downregulated, and cancer cells’ invasion and metastasis ability is decreased. The expression of CyclinD1 and c-Myc decreased, and the proliferation of nephroblastoma cells was inhibited. And the expression of inflammatory factors IL6 and IL8 was down-regulated, which inhibited the occurrence and development of nephroblastoma.

CCT4 is a protein-coding gene. The synergistic effect of Prefoldin and TriC/CCT on actin and tubulin folding and chaperone-mediated protein folding is its related pathway. GO annotations related to this gene include RNA binding and unfolded protein binding. A component of the T complex (TRiC) containing chaperone proteins is a molecular chaperone complex that helps proteins fold when ATP is hydrolyzed.^[18]^ The TRiC complex mediates the folding of WRAP53/TCAB1, which regulates telomere maintenance. As part of the TRiC complex, it may play a role in the assembly of BBSome. The BBSome complex participates in cilia formation and regulates the transport of vesicles to cilia.^[19]^ TRiC complex plays a role in the folding of actin and tubulin.

CCT4 may be involved in the cell cycle, promote apoptosis, and may be involved in the occurrence and development of nephroblastoma through the ERBB signal pathway. In cancer, the complexity of the cell cycle regulation mechanism and the frequency of component disorder reflect the importance of unplanned division to the malignant phenotype. The ability to maintain unplanned proliferation is a sign of cancer. The standard process of cell division occurs through the cell cycle, which consists of a series of highly regulated steps carefully planned at the molecular level by specific cyclins that bind to cyclin-dependent kinases.^[20]^ The universal mitogen-activated protein kinase (MAPK) signal transduction pathway is shared by 4 different cascades and named according to the composition of its MAPK layer. They are ERK1/2, JNK1/2/3, p38-MAPK, and ERK5 families, respectively.^[21]^ These signaling pathways exist in eukaryotic cells and play a key role in various cellular functions, and it is closely related to the ERBB signal pathway. MAPK and ERBB pathways are the oldest signal transduction pathways, which are widely used in the evolution of many physiological processes.

All eukaryotic cells have multiple ERBB signaling pathways that coordinate the regulation of gene expression, mitosis, metabolism, motility, survival, apoptosis, and differentiation.^[22]^ Maladjustment or overactivation of the ERBB signaling pathway usually leads to various diseases.^[23]^ Many studies have shown that the changes in the ERBB signaling pathway are significantly related to the occurrence of melanoma, breast cancer, esophageal cancer, colon cancer, gastric cancer, liver cancer, and other tumors.^[24]^ The mechanism leading to the occurrence and development of these cancers can be traced back to mutations in the upstream targets of the ERBB signal pathway.^[25]^ When the ERBB signaling pathway is activated, ERK1/2 regulates various transcription factors, which regulate behaviors involved in fundamental cellular processes, including proliferation, differentiation, survival, and motility. About 2/3 of human cancers, include skin, colon, lung, and pancreatic cancer.^[26]^ Studies have shown that in tumor patients, the activation of the ERBB signal pathway and the upregulation of its related genes can promote the transformation of normal cells into tumor cells. In contrast, inhibition of the ERBB signal pathway can restore tumor cells to a non-transformed state in vitro and inhibit the growth of tumor cells in vivo.^[27]^ Therefore, the enhanced activation of the ERBB signaling pathway is closely related to the occurrence and development of nephroblastoma.

Reiss-mann et al^[28]^ pointed out that the affinity of CCT4 and CCT5 subunits to ATP is higher than that of other subunits, which can drive the asymmetric dynamic stroke of ATP hydrolysis, thus promoting the folding cycle. Many studies have shown that CCT family genes are closely related to the occurrence and development of tumors. For example, there is a significant difference in the expression of TCP1/CCT2/CCT3/CCT4/CCT5/CCT6A/CCT7/CCT8 in hepatocellular carcinoma. The overexpression of TCP1/CCT2/CCT3/CCT4/CCT5/CCT6A is related to the abnormal regulation of the Myc target gene, hypoxia-inducible factor target gene, and cell cycle, especially the G1max S transition.^[29]^ In blastoma, the expression mutation of CCT family genes, in which CCT6A has the most incredible expression mutation and amplification induction in blastoma, has a negative correlation with survival rate.^[30]^

CCT4 interacts with several subunits of pre-folded protein (PFDN). PFDN, which consists of 6 subunits, is a hexamer chaperone protein complex. The expression of multiple subunits of PFDN is upregulated in tumors.^[31]^ PFDN is mainly involved in the cytoskeleton assembly during the folding of actin and tubulin monomers. Some literature studies have shown that the expression of PFDN1 positively correlates with tumor size and invasion. At the same time, silencing PFDN1 can lead to cell cycle dysfunction of G2amp M, which can inhibit tumor proliferation and movement. In addition, overexpression of PFDN1 induces tumor growth, metastasis, cell invasion, and epithelial-mesenchymal transformation.^[32]^ Therefore, it is speculated that the low expression of CCT4 promotes the development of nephroblastoma by regulating cell proliferation.

Although this paper has conducted rigorous bioinformatics analysis, there are still some shortcomings. In this study, no clinical samples were tested to verify the function of CCT4 in nephroblastoma further. Therefore, in future research, we should explore this aspect in depth.

In conclusion, this study used bioinformatics methods to examine the data from a large number of nephroblastoma samples and discovered that CCT family genes, particularly CCT4, play a crucial role in the occurrence and progression of nephroblastoma and may do so via the ERBB signal pathway. CCT4 might be a promising nephroblastoma biomarker. However, the mechanism and clinical application value of CCT4 in nephroblastoma need to be further verified.

## Author contributions

**Conceptualization:** Xiaochen Ni.

**Data curation:** Xiaochen Ni.

**Formal analysis:** Haoyuan Wang.

**Investigation:** Jianzhi Su.

**Methodology:** Jianzhi Su.

**Project administration:** Bin Liu.

**Resources:** Bin Liu.

**Software:** Bin Liu.

**Supervision:** Lei Zhang.

**Validation:** Lei Zhang.

**Visualization:** Haoyuan Wang.

**Writing – original draft:** Haoyuan Wang.
